# Supplementary figures and images for: Eldecalcitol is more effective in promoting osteogenesis than alfacalcidol in Cyp27b1-knockout mice
Source: PLoS One. 2018 Oct 3;13(10):e0199856. doi: 10.1371/journal.pone.0199856 (PMC6169848; doi:10.1371/journal.pone.0199856)

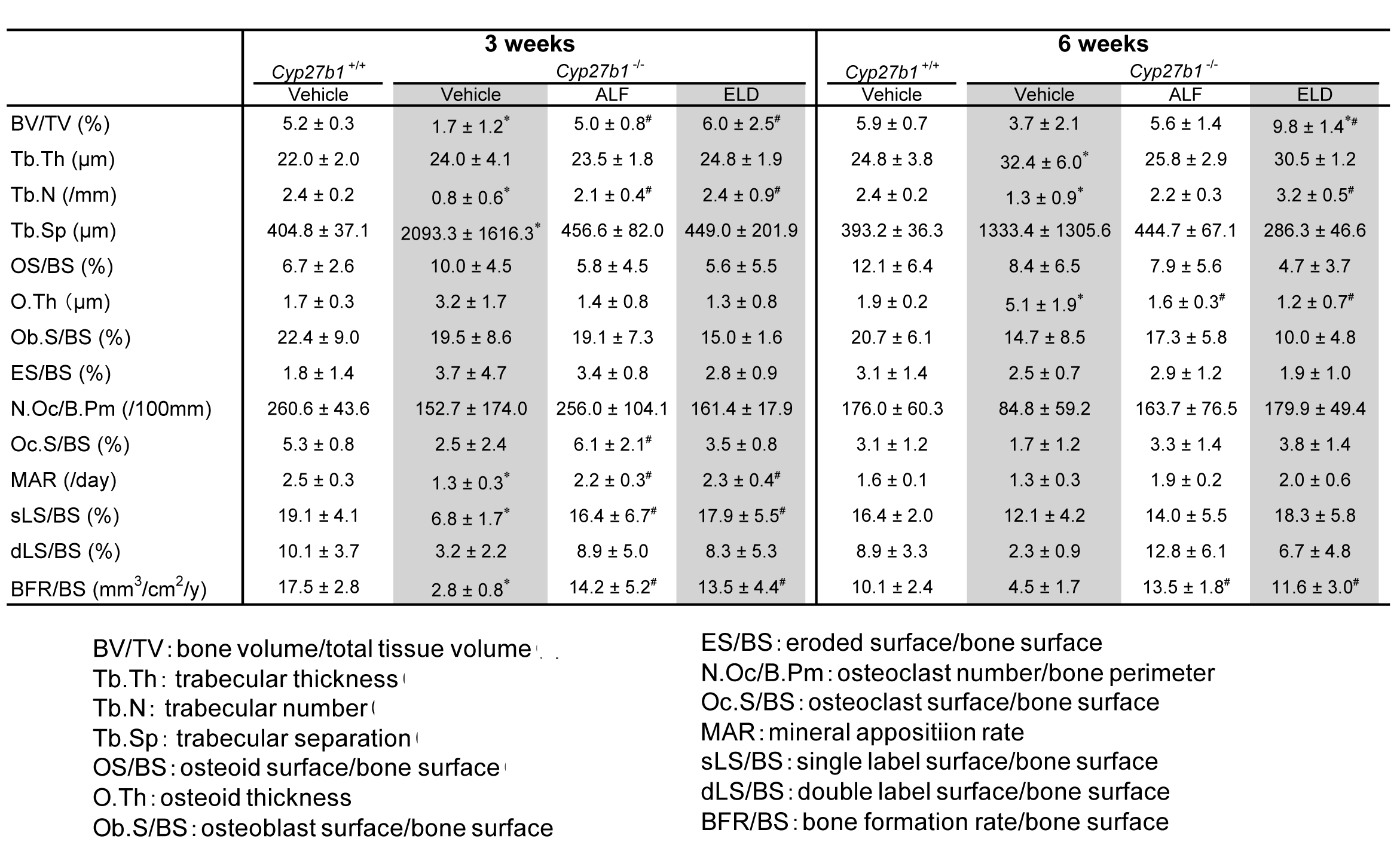

Supplement: S1 Table — *P < 0.05 vs. Cyp27b1+/+ mice administered vehicle, Student’s t-test. #P < 0.05 vs. Cyp27b1–/–mice administered vehicle, Dunnett’s test. (TIF) [file pone.0199856.s001.tif]
